# *PLAG1* Promotes Proliferation and Differentiation of Sujiang Pig MuSCs: Insights from Whole-Transcriptome and ceRNA Network Analyses

**DOI:** 10.3390/vetsci13080734

**Published:** 2026-07-24

**Authors:** Li Zhang, Hongxia Li, Anqi Dou, Changyao Fu, Suyi Sun, Shinuo Cao, Qingkang Zhou, Wei Miao, Mo Zhou, Wenhao Wang, Jialong Xu, Shanyuan Zhu

**Affiliations:** 1 Engineering Technology Research Center for Modern Animal Science and Novel Veterinary Pharmaceutic Development, Jiangsu Key Laboratory for High-Tech Research and Development of Veterinary Biopharmaceuticals, Jiangsu Agri-Animal Husbandry Vocational College, Taizhou 225300, China; lzhang@jsahvc.edu.cn (L.Z.); lhx92@jsahvc.edu.cn (H.L.); shinuo_cao@jsahvc.edu.cn (S.C.); zhouqingkang1220@126.com (Q.Z.); mo_zhou@jsahvc.edu.cn (M.Z.); 2 School of Biopharmaceuticals, Jiangsu Agri-Animal Husbandry Vocational College, Taizhou 225300, China; 2300790303@jsahvc.edu.cn (A.D.); 2300790304@jsahvc.edu.cn (C.F.); 2300790314@jsahvc.edu.cn (S.S.); 2021010402@jsahvc.edu.cn (W.M.); 3 Jiangsu Jiangquhai Pig Breeding Co., Ltd., Taizhou 225300, China; wwh11@126.com; 4 Medical School, Nanjing University, Nanjing 210093, China

**Keywords:** PLAG1, skeletal muscle satellite cells, RNA sequencing, ceRNA network, Sujiang pig

## Abstract

Skeletal muscle satellite cells (MuSCs) are essential for muscle growth and development, but their regulatory mechanisms remain unclear. This study aimed to characterize the expression pattern of pleomorphic adenoma gene 1 (*PLAG1*), determine its effects on MuSC proliferation and differentiation, and explore its potential regulatory mechanisms through integrated transcriptomic and ceRNA analyses in Sujiang pig MuSCs. Results showed that *PLAG1* was widely expressed in multiple porcine tissues and was significantly upregulated during MuSC differentiation. *PLAG1* overexpression promoted MuSC proliferation and differentiation, whereas *PLAG1* knockdown produced the opposite effects. Whole-transcriptome sequencing following *PLAG1* knockdown identified 432 differentially expressed mRNAs, 70 miRNAs, 163 lncRNAs, and 150 circRNAs. Enrichment analysis revealed that differentially expressed mRNAs were mainly associated with cell cycle regulation, DNA replication, and the p53 signaling pathway. Western blot analysis further showed that *PLAG1* knockdown reduced CDK1 and PCNA protein levels. ceRNA network analysis revealed potential regulatory associations among differentially expressed lncRNAs, circRNAs, miRNAs, and mRNAs, and the expression changes in selected differentially expressed RNAs were further validated by qRT-PCR. Collectively, these findings indicate that *PLAG1* promotes MuSC proliferation and differentiation and may be associated with cell cycle-related gene expression changes and potential non-coding RNA regulatory networks in porcine MuSCs.

## Simple Summary

Pork is an important source of animal protein worldwide, and improving skeletal muscle growth is critical for enhancing pork production. Skeletal muscle growth largely depends on the proliferation and differentiation of skeletal muscle satellite cells (MuSCs). In this study, we investigated the role of *PLAG1* in skeletal muscle satellite cells isolated from the Chinese indigenous Sujiang pig. Our results showed that *PLAG1* promotes both cell proliferation and differentiation. Further transcriptome analyses indicated that *PLAG1* may regulate these processes through cell cycle-related genes and a competing endogenous RNA (ceRNA) regulatory network. These findings provide mechanistic insights into skeletal muscle development and identify candidate regulatory targets for pig breeding.

## 1. Introduction

Meat production performance in pigs is closely associated with skeletal muscle growth and development, a process that primarily depends on the proliferation, differentiation, and fusion of skeletal muscle satellite cells (MuSCs). MuSCs are a population of adult stem cells located between the basal lamina and the sarcolemma of muscle fibers and typically remain in a quiescent state under physiological conditions. Following muscle injury, MuSCs are rapidly activated and re-enter the cell cycle after a period of cellular growth. Subsequently, they proliferate as myoblasts and undergo terminal differentiation and fusion to form multinucleated myotubes and new muscle fibers, thereby contributing to muscle regeneration and growth [1,2,3].

The proliferation and differentiation of skeletal muscle satellite cells are tightly controlled by multilayered gene regulatory networks involving transcriptional, post-transcriptional, and epigenetic regulation. Among these regulatory mechanisms, transcription factors and classical signaling pathways play pivotal roles in determining satellite cell fate [4]. MicroRNAs (miRNAs), long non-coding RNAs (lncRNAs), and circular RNAs (circRNAs) can modulate target gene expression through post-transcriptional regulatory mechanisms and interact with one another through competing endogenous RNA (ceRNA) networks, thereby forming complex gene regulatory systems [5,6]. Among these molecules, miRNAs are well-established post-transcriptional regulators that play crucial roles in myoblast proliferation and differentiation [7]. Muscle-specific miRNAs (myomiRs), including miR-1 [8], miR-133 [9,10], and miR-206 [11], have been shown to regulate myogenic differentiation and proliferation. In addition to miRNAs, lncRNAs also play important regulatory roles in skeletal muscle growth and development [12,13,14,15]. Meanwhile, accumulating evidence has demonstrated that circRNAs are involved in the regulation of skeletal muscle growth and development [16,17]. Furthermore, these non-coding RNAs can competitively bind miRNAs to form ceRNA regulatory networks, thereby coordinately regulating gene expression at the post-transcriptional level [18]. However, systematic studies investigating ceRNA networks in porcine skeletal muscle satellite cells remain limited, and the molecular mechanisms through which specific regulatory factors mediate these networks have yet to be fully elucidated.

Pleomorphic adenoma gene 1 (*PLAG1*) encodes a typical C2H2 zinc-finger transcription factor and belongs to the PLAG family of transcription factors, which also includes pleomorphic adenoma gene-like 1 (*PLAGL1*) and pleomorphic adenoma gene-like 2 (*PLAGL2*) [19]. Studies have shown that *PLAG1* can promote cell proliferation through the regulation of insulin-like growth factor 2 (*IGF2*) expression [19]. In livestock species, *PLAG1* has been reported to be significantly associated with economically important traits, including body size, body weight, and growth rate, and is considered one of the key candidate genes influencing growth and development in cattle and pigs [20,21]. However, studies investigating the role of *PLAG1* in skeletal muscle development remain limited, and its functions and underlying molecular mechanisms in the proliferation and differentiation of skeletal muscle satellite cells have not yet been fully elucidated.

The Sujiang pig is a Chinese indigenous pig breed characterized by superior meat quality and abundant genetic resources [22]. In our previous single-trait and multi-trait genome-wide association studies (GWASs) conducted in a Sujiang pig population, the genomic region containing *PLAG1* was found to harbor multiple single-nucleotide polymorphisms (SNPs) significantly associated with backfat thickness and chest circumference [21]. Further haplotype analysis revealed the presence of specific haplotype structures within this region, some of which were significantly associated with lower backfat thickness and larger chest circumference. These findings suggest that *PLAG1* may play an important regulatory role in skeletal muscle development and the formation of growth-related traits in pigs through its effects on growth and fat deposition.

In the present study, we investigated the regulatory functions of *PLAG1* in porcine skeletal muscle satellite cells. We first characterized the evolutionary conservation, structural features, and expression patterns of *PLAG1*, and then evaluated its effects on MuSC proliferation and differentiation through overexpression and knockdown experiments. Furthermore, integrated transcriptomic and ceRNA analyses following *PLAG1* knockdown were performed to explore its potential regulatory mechanisms. This study may provide a basis for further understanding the role of *PLAG1* in MuSC regulation.

## 2. Materials and Methods

### 2.1. Animals and Tissue Collection

All animal procedures were conducted in accordance with the Guide for the Care and Use of Laboratory Animals issued by the Ministry of Science and Technology of China and were approved by the Animal Care and Management Committee of Jiangsu Agri-Animal Husbandry Vocational College (Approval No. jsahvc-2024-78; approved on 10 January 2024).

The Sujiang pigs used in this study were obtained from Jiangsu Jiangquhai Pig Breeding Co., Ltd. (Taizhou, China). Three healthy female Sujiang pigs aged 11 days were included in the study. Each pig was considered an independent experimental unit and biological replicate, resulting in three independent biological replicates (*n* = 3). Samples from each pig were handled separately throughout tissue collection, skeletal muscle satellite cell isolation and culture, and subsequent analyses. Tissue samples including the longissimus dorsi muscle, heart, liver, spleen, lung, kidney, and adipose tissue were collected immediately. A portion of the longissimus dorsi muscle from each pig was used for the isolation and culture of skeletal muscle satellite cells, whereas the remaining tissue samples were rapidly frozen in liquid nitrogen and stored at −80 °C for subsequent RNA extraction.

### 2.2. Isolation, Culture, and Differentiation of Skeletal Muscle Satellite Cells

Porcine skeletal muscle satellite cells were isolated from the longissimus dorsi muscle of the 11-day-old Sujiang pigs described above. Briefly, the muscle samples were minced and digested at 37 °C for 1 h in high-glucose DMEM (Gibco, Grand Island, NY, USA) containing 2 mg/mL Dispase II and 4 mg/mL collagenase II (Sigma-Aldrich, St. Louis, MO, USA). After sequential filtration through 100 μm and 40 μm cell strainers (Solarbio Science, Beijing, China) and erythrocyte lysis (Solarbio Science, Beijing, China), the isolated cells were seeded in Matrigel-coated culture dishes (BD Biosciences, Bedford, MA, USA). Cells were maintained at 37 °C with 5% CO_2_ in DMEM/F12 (HyClone, Logan, UT, USA) supplemented with 20% fetal bovine serum (Gemini Bio-Products, West Sacramento, CA, USA), 1% penicillin–streptomycin (Solarbio Science, Beijing, China), and 5 ng/mL basic fibroblast growth factor (PeproTech, Cranbury, NJ, USA). Myogenic differentiation was induced by replacing the growth medium with high-glucose DMEM containing 2% horse serum (Gibco, Grand Island, NY, USA), as previously described [23].

### 2.3. Bioinformatic Analysis of PLAG1

To systematically characterize the biological features of *PLAG1*, the *PLAG1* mRNA sequences of 13 species were retrieved from the NCBI database (https://www.ncbi.nlm.nih.gov/nucleotide/; accessed on 21 December 2025) (Table S1), including *Homo sapiens* (human), *Sus scrofa* (pig), *Mus musculus* (house mouse), *Bos taurus* (domestic cattle), *Ovis aries* (sheep), *Capra hircus* (goat), *Gallus gallus* (chicken), *Rattus norvegicus* (Norway rat), *Macaca mulatta* (rhesus monkey), *Anas platyrhynchos* (mallard), *Anser cygnoides* (swan goose), *Xenopus tropicalis* (tropical clawed frog), and *Danio rerio* (zebrafish). The corresponding amino acid sequences were obtained by open reading frame (ORF) translation, and a phylogenetic tree was constructed using the Neighbor-Joining method implemented in MEGA v11.0.13 (https://www.megasoftware.net/; accessed on 21 December 2025).

The three-dimensional structure of the porcine PLAG1 protein was predicted by homology modeling using the SWISS-MODEL web server (https://swissmodel.expasy.org/; accessed on 23 December 2025). Among the generated models, the structure with the highest QSQE and GMQE scores was selected for subsequent analyses. Visualization and structural characterization of the predicted PLAG1 protein were performed using PyMOL v3.1.6.1 (https://www.pymol.org/; accessed on 23 December 2025).

### 2.4. RNA Extraction and qRT-PCR Analysis

Total RNA was extracted from tissues and cells using TRIzol reagent (Life Technologies, Carlsbad, CA, USA). For mRNA expression analysis, first-strand cDNA was synthesized using the EasyScript One-Step gDNA Removal and cDNA Synthesis SuperMix kit (TransGen Biotech, Beijing, China) according to the manufacturer’s instructions. Quantitative real-time PCR (qRT-PCR) was performed using PerfectStart Green qPCR SuperMix (TransGen Biotech, Beijing, China) following the manufacturer’s protocol. The 18S rRNA gene was used as the internal reference. The primer sequences used for mRNA analysis were as follows: *PLAG1*-F, 5′-AATTACAAAGGCACATGGCTACCC-3′; *PLAG1*-R, 5′-CCGCACTCTTCGCACTTGAAC-3′; *MAP3K8*-F, 5′-CATGGTTGTCGTCGGTCAGA-3′; *MAP3K8-R*, 5′-ATCTGATAGCGCCCGTTCTG-3′; *STAT1*-F, 5′-CGGTCTTATTCCCTGGACGA-3′; *STAT1*-R, 5′-AGCCCACAATACACCCATCA-3′; *HGF*-F, 5′-CCATTCACATGCAAGGCCTT-3′; *HGF*-R, 5′-ACCGTTCCCTTATAGCTACCTC-3′; 18S-F, 5′-CCTGCGGCTTAATTTGACTC-3′; and 18S-R, 5′-ATGCCAGAGTCTCGTTCGTT-3′.

For miRNA expression analysis, first-strand cDNA was synthesized using the First Strand cDNA Synthesis Kit (Invitrogen, Carlsbad, CA, USA), according to the manufacturer’s instructions. qRT-PCR was performed using the QuantiNova SYBR Green PCR Kit (QIAGEN, Hilden, Germany), following the manufacturer’s protocol. U6 snRNA was used as the internal reference. The primer sequences used for miRNA analysis were as follows: U6-F, 5′-CTCGCTTCGGCAGCACA-3′; U6-R, 5′-AACGCTTCACGAATTTGCGT-3′; miR-532-y-F, 5′-GCCTCCCACACCCAAGG-3′; and miR-671-y-F, 5′-CGTCCGGTTCTCAGGGC-3′.

### 2.5. PLAG1 Overexpression and Knockdown

The *PLAG1* overexpression plasmid (PLVX-IRES-ZsGreen1-*PLAG1*) and the control plasmid (PLVX-IRES-ZsGreen1-Scramble) were constructed by Azenta Life Sciences (Suzhou, China). Lentiviral particles were subsequently packaged in HEK293T cells (Shanghai Beiweimin Technology Co., Ltd., Shanghai, China). For lentiviral packaging, the overexpression or control plasmid was co-transfected with the packaging plasmids pSPAX2 and pMD2.G into HEK293T cells at a mass ratio of 3:2:1 using PolyJet in vitro DNA transfection reagent (SignaGen Laboratories, Rockville, MD, USA). At 72 h post-transfection, the viral supernatant was collected and concentrated using Amicon Ultra 100 kDa MWCO filters (100 kDa MWCO, Merck KGaA, Darmstadt, Germany). The concentrated viral particles were stored at −80 °C for subsequent cell infection.

The design, construction, and packaging of lentiviral vectors for *PLAG1* knockdown were performed by MIJIA Biotechnology Co., Ltd. (Beijing, China). Three shRNAs targeting *PLAG1* (sh*PLAG1*#1, sh*PLAG1*#2, and sh*PLAG1*#3) were designed, with target sequences of 5′-GACTTCTCCCAGTTGTTTAAT-3′, 5′-CTGTGTGACAAGGCCTTTAAC-3′, and 5′-CACCACCTGTCCTTCAAATAC-3′, respectively. The sequence 5′-CCTAAGGTTAAGTCGCCCTCG-3′ was used as the scramble control. These oligonucleotide sequences were cloned into the pLV-shRNA-EGFP-Puro vector and packaged into lentiviral particles.

To achieve *PLAG1* overexpression or knockdown in porcine MuSCs, cells at approximately 80% confluence were infected with lentivirus at a multiplicity of infection (MOI) of 3. At 72 h post-infection, cells were harvested, and *PLAG1* mRNA expression levels were determined by qRT-PCR to evaluate the efficiency of overexpression or knockdown.

### 2.6. Cell Proliferation Assays

#### 2.6.1. CCK-8 Assay

Cell proliferation was evaluated using an Enhanced Cell Counting Kit-8 (Beyotime Biotechnology, Shanghai, China). Briefly, porcine MuSCs subjected to different treatments were seeded into 96-well plates at approximately 4 × 10^3^ cells per well, with six replicate wells per group. After cell attachment, 10 μL of CCK-8 reagent was added to each well at 0, 12, 24, 48, 72, and 96 h, followed by incubation for an additional 2 h. Absorbance was measured at 450 nm using a Model 680 Microplate Reader (Bio-Rad Laboratories, Inc., Hercules, CA, USA). Cell proliferation curves were generated based on the absorbance values obtained at each time point.

#### 2.6.2. EdU Assay

DNA synthesis was assessed using a BeyoClick EdU-555 Cell Proliferation Kit (Beyotime Biotechnology, Shanghai, China). Briefly, porcine MuSCs subjected to different treatments were seeded into 24-well plates. When the cells reached an appropriate density, EdU was added to a final concentration of 10 μM and incubated for 2 h. The cells were then fixed with 4% paraformaldehyde for 15 min, washed with PBS, and permeabilized with 0.5% Triton X-100 for 10 min. EdU-positive cells were labeled using the Click reaction, according to the manufacturer’s instructions, and cell nuclei were counterstained with DAPI.

### 2.7. Myogenic Differentiation Assay

Myotube formation was evaluated by immunofluorescence staining using a mouse anti-myosin heavy chain antibody (MYHC, 1:100, Developmental Studies Hybridoma Bank, Iowa City, IA, USA). Differentiated myotubes were fixed with 4% paraformaldehyde (Solarbio Science, Beijing, China), and blocked with blocking buffer (Beyotime Biotechnology, Shanghai, China). The differentiated myotubes were then incubated with the primary antibody against MYHC, followed by incubation with an Alexa Fluor 488-conjugated goat anti-mouse IgG secondary antibody (1:400, Thermo Fisher Scientific, Waltham, MA, USA). Nuclei were counterstained with DAPI (Beyotime Biotechnology, Shanghai, China), and images were captured using an Axio Vert.A1 inverted fluorescence microscope (Carl Zeiss Microscopy GmbH, Jena, Germany).

### 2.8. Western Blot Analysis

Cells from the *PLAG1* knockdown group and the shNC control group were collected after 3 days of differentiation. Total proteins were extracted, separated by SDS-PAGE, and transferred onto PVDF membranes. The membranes were incubated with primary antibodies against CDK1 Rabbit Monoclonal Antibody (Beyotime Biotechnology, Shanghai, China; 1:1000), PCNA Rabbit Monoclonal Antibody (Beyotime Biotechnology, Shanghai, China; 1:1000), and GAPDH Mouse Monoclonal Antibody (Beyotime Biotechnology, Shanghai, China; 1:5000), followed by incubation with HRP-conjugated anti-rabbit and anti-mouse secondary antibodies (Beyotime Biotechnology, Shanghai, China; 1:5000). GAPDH was used as the loading control. Relative protein levels were quantified using Fiji ImageJ software (version 1.54g), and protein expression levels were first normalized to GAPDH and then further normalized relative to the shNC control group.

### 2.9. RNA Extraction, Library Construction, and Sequencing

Cells from the *PLAG1* knockdown group and the control group were collected after 3 days of differentiation, with three biological replicates per group. Total RNA was extracted using TRIzol Reagent. RNA concentration and purity were determined using a NanoDrop 2000 spectrophotometer (Thermo Fisher Scientific, Waltham, MA, USA), and RNA integrity was assessed using an Agilent 2100 Bioanalyzer (Agilent Technologies, Santa Clara, CA, USA). Qualified RNA samples were submitted to Genedenovo Biotechnology Co., Ltd. (Guangzhou, China) for whole-transcriptome sequencing.

For mRNA, lncRNA, and circRNA library construction, rRNA was first removed using the Ribo-off rRNA Depletion Kit (Vazyme, Nanjing, China). Strand-specific libraries were then prepared using the VAHTS Universal V6 RNA-seq Library Prep Kit for Illumina (Vazyme, Nanjing, China). Following RNA fragmentation, first-strand cDNA was synthesized, and strand specificity was achieved during second-strand synthesis using a strand-specific enzymatic method. The resulting double-stranded cDNA was subjected to end repair, A-tailing, and adaptor ligation, followed by PCR amplification and bead purification. Qualified libraries were sequenced on the NovaSeq X Plus platform (Illumina, Inc., San Diego, CA, USA) using the PE150 paired-end mode.

For miRNA library construction, RNA fragments of 18–30 nt were isolated from total RNA by agarose gel electrophoresis and used for library preparation with the Small RNA Library Prep Kit (NEB, Ipswich, MA, USA). Adapter-ligated small RNAs were reverse-transcribed and PCR-amplified. Approximately 140 bp fragments were recovered by agarose gel purification to generate sequencing libraries, which were subsequently sequenced on the same NovaSeq X Plus platform.

### 2.10. Transcriptome Data Analysis

Raw sequencing data were processed by removing adaptor sequences and low-quality reads to obtain clean reads. The clean reads were aligned to the porcine reference genome Sscrofa11.1 (Ensembl release 112, GCA_000003025.6) using HISAT2 (v2.1.0; http://daehwankimlab.github.io/hisat2/; accessed on 26 April 2025). Gene expression levels were quantified using fragments per kilobase of transcript per million mapped reads (FPKM), and differential expression analysis was performed using DESeq (v1.20.0; Bioconductor). The criteria for identifying differentially expressed transcripts were as follows: mRNAs, |log_2_FC| > 1 and FDR < 0.05; miRNAs and circRNAs, |log_2_FC| > 1 and *p* < 0.05; lncRNAs, |log_2_FC| > log_2_(1.5) and *p* < 0.05.

Gene Ontology (GO) enrichment analysis was performed using the GO.db R package (v3.14.0; https://www.bioconductor.org/packages/release/data/annotation/html/GO.db.html; accessed on 18 May 2025). Kyoto Encyclopedia of Genes and Genomes (KEGG) pathway enrichment analysis was conducted using KEGG Release 101 (http://www.kegg.jp; accessed on 20 May 2025). Gene Set Enrichment Analysis (GSEA) was performed using GSEA software (v2.2.4; http://software.broadinstitute.org/gsea/index.jsp; accessed on 25 May 2025). ceRNA regulatory networks were constructed based on lncRNA–miRNA–mRNA and circRNA–miRNA–mRNA interaction pairs according to the ceRNA hypothesis and previous studies [24]. Potential regulatory relationships were further screened based on expression correlations and visualized using Cytoscape (v3.6.0; https://cytoscape.org/; accessed on 11 March 2026). The raw sequencing data generated in this study have been deposited in the NCBI Sequence Read Archive (SRA) database under BioProject accession number PRJNA1477753.

### 2.11. Statistical Analysis

Data are presented as mean ± SEM. Statistical analyses were performed using SPSS software (v26.0; https://www.ibm.com/spss; accessed on 30 December 2025). Data normality and homogeneity of variance were assessed using the Shapiro–Wilk test and Levene’s test, respectively. All datasets subjected to parametric analysis met the relevant assumptions. Comparisons between two independent groups, including the *PLAG1* overexpression group versus the NC control group and the sh*PLAG1* knockdown group versus the shNC control group, were performed using a two-tailed independent-samples Student’s *t*-test. Comparisons among multiple groups, including the shNC control group and different sh*PLAG1* knockdown groups (sh*PLAG1*#1, sh*PLAG1*#2, sh*PLAG1*#3, and sh*PLAG1*#123), were performed using one-way analysis of variance (ANOVA), followed by Dunnett’s multiple-comparison test. CCK-8 time-course data were analyzed using two-way ANOVA, followed by Sidak-adjusted pairwise comparisons between the treatment groups at each time point. A *p*-value < 0.05 was considered statistically significant.

## 3. Results

### 3.1. Evolutionary Conservation, Structural Features, and Expression Patterns of PLAG1 in Sujiang Pigs

**Figure 1:**
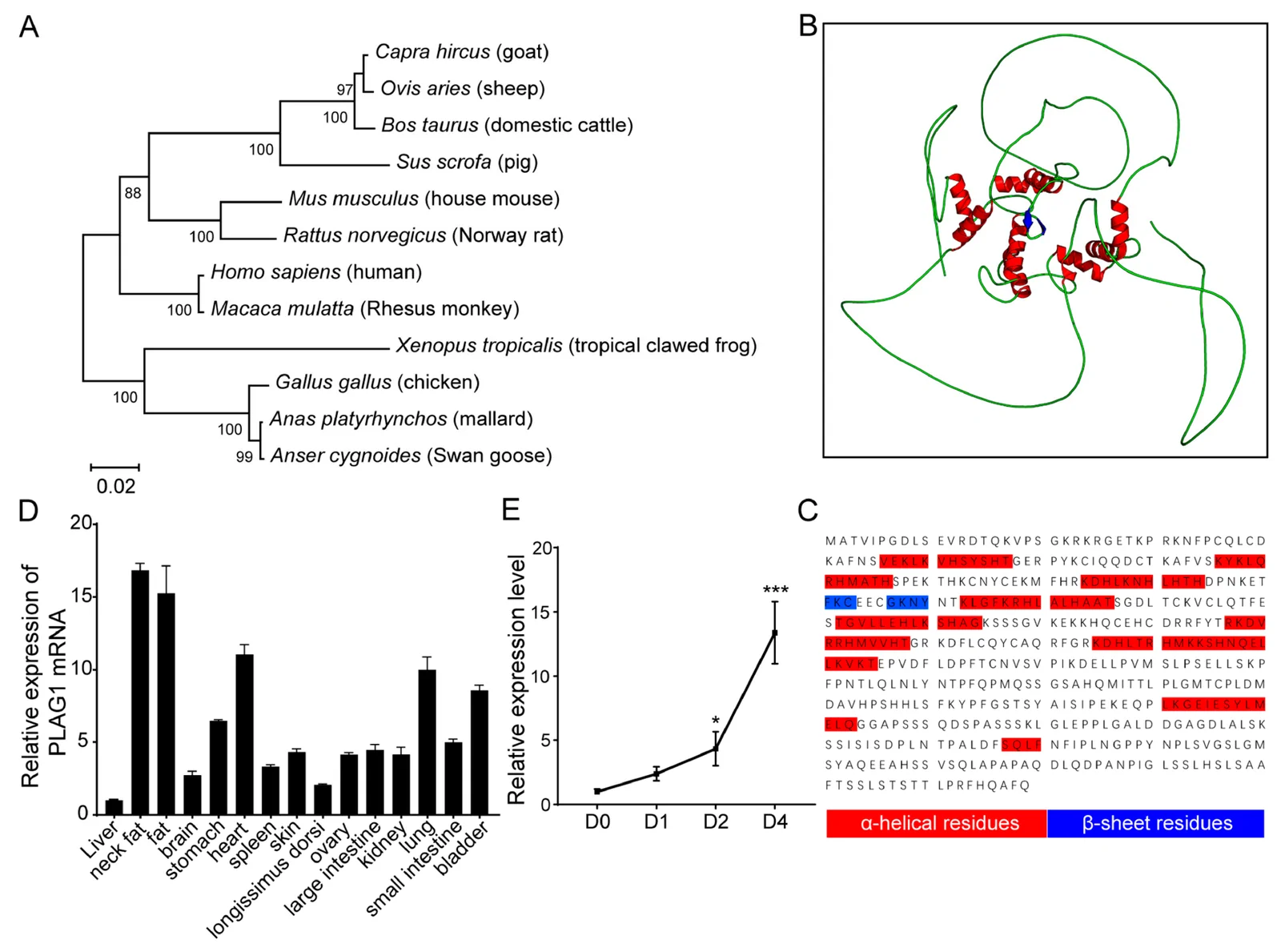
Phylogenetic analysis, structural prediction, and expression profile of PLAG1. (**A**) Phylogenetic analysis of PLAG1. A phylogenetic tree was constructed based on the amino acid sequences of PLAG1 from different species. Numbers at the branch nodes indicate bootstrap values, and the scale bar represents genetic distance. (**B**) Predicted three-dimensional structure of the porcine PLAG1 protein. The three-dimensional structure was predicted using homology modeling with SWISS-MODEL. α-Helices are shown in red, and β-sheets are shown in blue. (**C**) Amino acid sequence of the porcine PLAG1 protein. The protein consists of 499 amino acid residues. Predicted α-helical residues are highlighted in red, whereas β-sheet residues are highlighted in blue. (**D**) Tissue expression profile of *PLAG1*. Relative expression levels of *PLAG1* in different tissues of Sujiang pigs were determined by qRT-PCR using 18S rRNA as the reference gene. (**E**) Expression pattern of *PLAG1* during skeletal muscle satellite cell differentiation. Relative expression levels of *PLAG1* were determined by qRT-PCR at different stages of differentiation (D0, D1, D2, and D4) using 18S rRNA as the reference gene, with the D0 group serving as the control. Data are presented as mean ± SEM (*n* = 3). * *p* < 0.05, *** *p* < 0.001.

To systematically characterize the biological features of *PLAG1*, its evolutionary conservation, protein structural features, and expression patterns were analyzed. To investigate the homology of the PLAG1 protein, a phylogenetic tree was constructed based on the amino acid sequences of PLAG1 from 13 species (Figure 1A). The results showed that PLAG1 proteins from mammals clustered into a distinct branch, with porcine PLAG1 showing the closest evolutionary relationship to those of cattle, sheep, and goats. In contrast, avian and amphibian species were located in more distant branches, indicating a high degree of evolutionary conservation of *PLAG1* among mammals. Subsequently, the three-dimensional structure of the porcine PLAG1 protein was predicted by homology modeling using the SWISS-MODEL server (Figure 1B,C). The predicted structure contained approximately nine α-helices (red) and two β-sheets (blue), together with several flexible loop regions.

qRT-PCR analysis showed that *PLAG1* was expressed in all examined tissues of Sujiang pigs, although its expression levels varied considerably among tissues (Figure 1D). Relatively high expression levels were detected in adipose tissue, heart, and lung, whereas *PLAG1* expression was also detectable in the longissimus dorsi muscle. These results indicate that *PLAG1* exhibits a broad but tissue-specific expression pattern and may participate in multiple physiological processes. The expression dynamics of *PLAG1* during the differentiation of porcine skeletal muscle satellite cells were further investigated. As shown in Figure 1E, *PLAG1* expression increased progressively during differentiation and was significantly upregulated at the later stage of differentiation, suggesting that *PLAG1* may play a regulatory role in the differentiation of skeletal muscle satellite cells.

### 3.2. PLAG1 Regulates the Proliferation and Differentiation of Skeletal Muscle Satellite Cells

**Figure 2:**
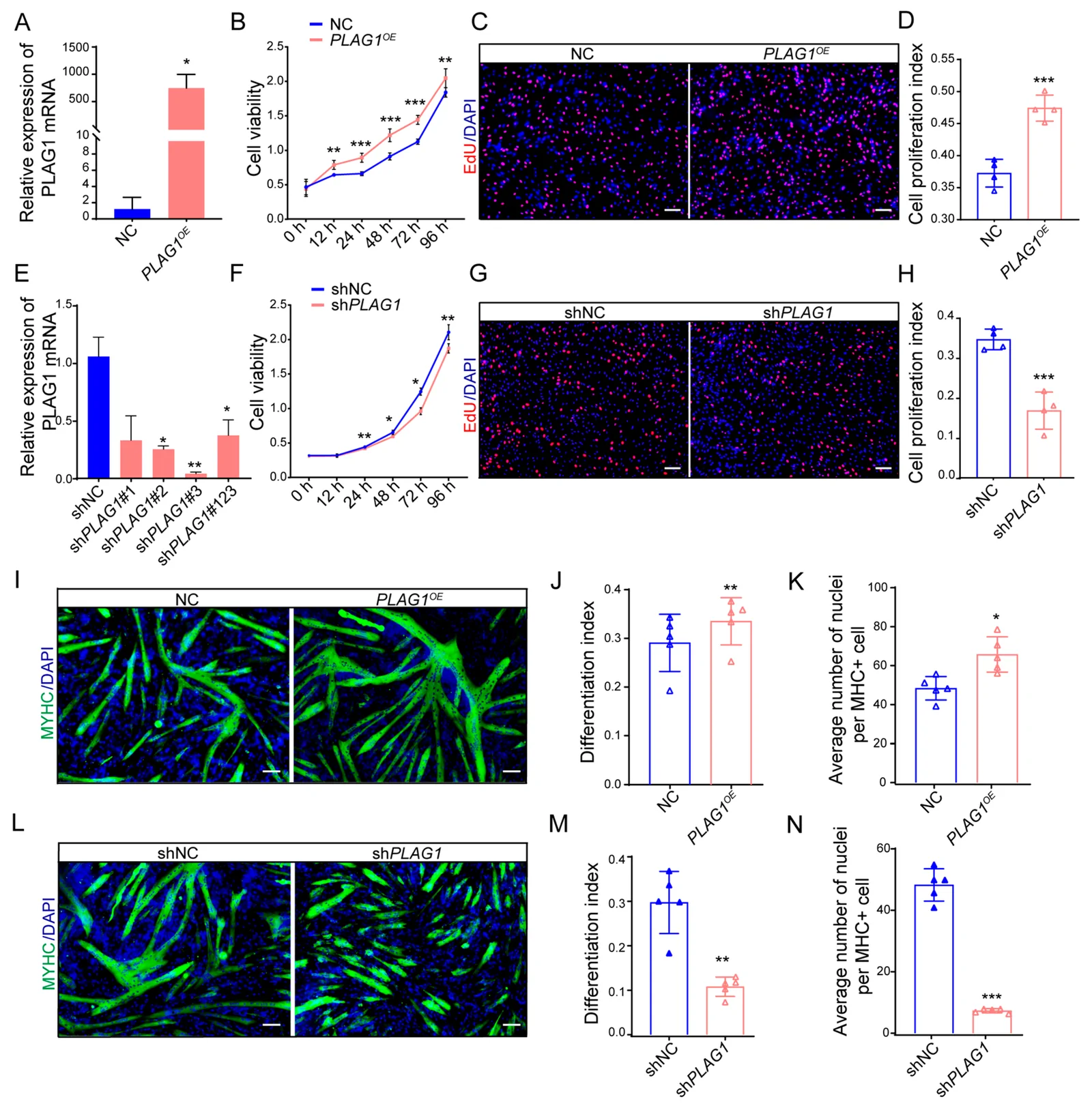
*PLAG1* overexpression and knockdown in Sujiang pig MuSCs. (**A**) qRT-PCR analysis of *PLAG1* overexpression efficiency. Relative expression levels were normalized to 18S rRNA and presented relative to the NC group (*n* = 3). (**B**) CCK-8 assay showing changes in cell viability following *PLAG1* overexpression (*n* = 6). (**C**) EdU immunofluorescence staining showing the effects of *PLAG1* overexpression on satellite cell proliferation. Red signals indicate EdU-positive nuclei, and blue signals indicate DAPI-stained nuclei. Scale bar = 100 μm. (**D**) Quantification of the percentage of EdU-positive cells following *PLAG1* overexpression (*n* = 4). (**E**) qRT-PCR analysis of the knockdown efficiencies of three shRNAs (sh*PLAG1*#1, sh*PLAG1*#2, and sh*PLAG1*#3) targeting *PLAG1*. Relative expression levels were normalized to 18S rRNA and presented relative to the shNC group (*n* = 3). (**F**) CCK-8 assay showing changes in cell viability following *PLAG1* knockdown (*n* = 4). (**G**) EdU immunofluorescence staining showing the effects of *PLAG1* knockdown on satellite cell proliferation. Red signals indicate EdU-positive nuclei, and blue signals indicate DAPI-stained nuclei. Scale bar = 100 μm. (**H**) Quantification of the percentage of EdU-positive cells following *PLAG1* knockdown (*n* = 4). (**I**) MYHC immunofluorescence staining showing the effects of *PLAG1* overexpression on satellite cell differentiation. MYHC (green) labels myotubes, and DAPI (blue) labels nuclei. Scale bar = 100 μm. (**J**) Quantification of the differentiation index following *PLAG1* overexpression. The differentiation index was calculated as the percentage of nuclei within myotubes containing at least two nuclei relative to the total number of nuclei (*n* = 5). (**K**) Average number of nuclei per MYHC-positive myotube following *PLAG1* overexpression (*n* = 5). (**L**) MYHC immunofluorescence staining showing the effects of *PLAG1* knockdown on satellite cell differentiation. MYHC (green) labels myotubes, and DAPI (blue) labels nuclei. Scale bar = 100 μm. (**M**) Quantification of the differentiation index following *PLAG1* knockdown (*n* = 5). (**N**) Average number of nuclei per MYHC-positive myotube following *PLAG1* knockdown (*n* = 5). The NC and shNC groups served as controls for the overexpression and knockdown experiments, respectively. Data are presented as mean ± SEM. * *p* < 0.05, ** *p* < 0.01, *** *p* < 0.001.

To investigate the role of *PLAG1* in the proliferation and differentiation of skeletal muscle satellite cells, both overexpression and knockdown experiments were performed. *PLAG1* overexpression was achieved through lentivirus-mediated gene transduction, with cells infected with PLVX-IRES-ZsGreen1-Scramble serving as the control group. Compared with the control group (NC), *PLAG1* expression was significantly increased in the *PLAG1* overexpression group (*PLAG1^OE^*) (Figure 2A), indicating successful overexpression. To achieve *PLAG1* knockdown, three shRNAs targeting *PLAG1* (sh*PLAG1*#1, sh*PLAG1*#2, and sh*PLAG1*#3) were designed and delivered into skeletal muscle satellite cells via lentiviral infection. Among the three shRNAs, sh*PLAG1*#3 exhibited the highest knockdown efficiency and was therefore selected for subsequent functional analyses (Figure 2E), with shNC serving as the negative control.

The effects of *PLAG1* on satellite cell proliferation were subsequently evaluated. CCK-8 analysis showed that *PLAG1* overexpression significantly increased cell viability (Figure 2B). Consistently, EdU staining and quantitative analysis revealed a significantly higher proportion of EdU-positive cells in the *PLAG1^OE^* group (Figure 2C,D). In contrast, *PLAG1* knockdown markedly inhibited satellite cell proliferation, as demonstrated by both the CCK-8 and EdU assays, which showed significantly reduced proliferative capacity in the knockdown group (Figure 2F–H).

The effects of *PLAG1* on satellite cell differentiation were further investigated. Myotube formation was assessed by MYHC immunofluorescence staining, and both the differentiation index (the percentage of nuclei within myotubes containing ≥2 nuclei relative to the total number of nuclei) and the average number of nuclei per MYHC-positive myotube were quantified. As shown in Figure 2I, *PLAG1* overexpression promoted cell fusion and resulted in the formation of larger myotubes. Correspondingly, the differentiation index was significantly increased (Figure 2J, *p* < 0.01), and the average number of nuclei per MYHC-positive myotube was also significantly elevated (Figure 2K, *p* < 0.05). Conversely, *PLAG1* knockdown resulted in the formation of noticeably smaller myotubes (Figure 2L), accompanied by a significant reduction in the differentiation index (Figure 2M, *p* < 0.01) and the average number of nuclei per MYHC-positive myotube (Figure 2N, *p* < 0.001). Taken together, these findings demonstrate that *PLAG1* positively regulates both the proliferation and differentiation of skeletal muscle satellite cells.

### 3.3. Identification of Differentially Expressed RNAs Following PLAG1 Knockdown

**Figure 3:**
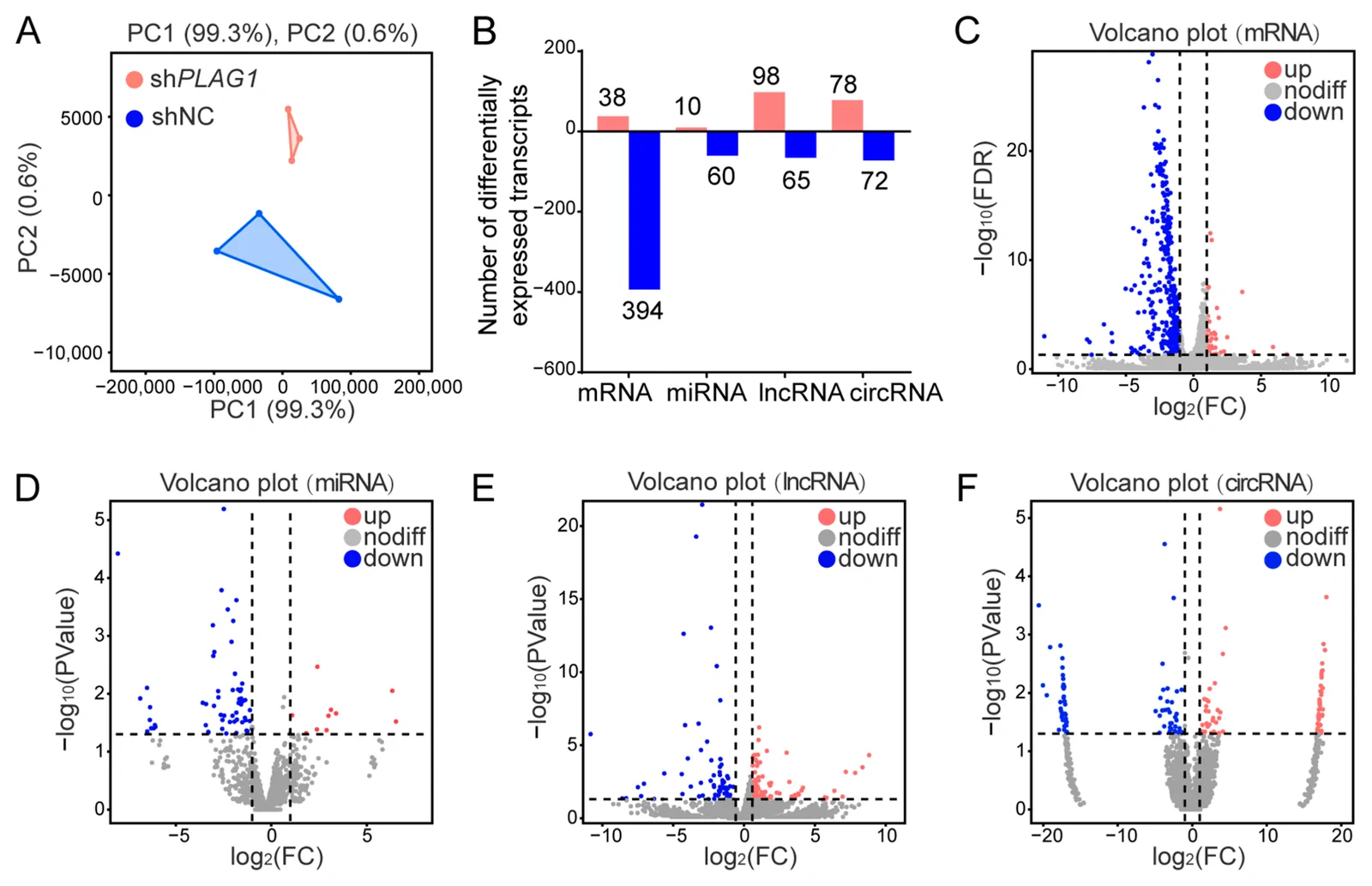
Differential expression analysis of the transcriptome sequencing data from skeletal muscle satellite cells following *PLAG1* knockdown. (**A**) Principal component analysis (PCA) plot. (**B**) Distribution of upregulated and downregulated differentially expressed transcripts, including differentially expressed mRNAs (DEmRNAs), miRNAs (DEmiRNAs), lncRNAs (DElncRNAs), and circRNAs (DEcircRNAs). (**C**–**F**) Volcano plots showing the differential expression profiles of DEmRNAs, DEmiRNAs, DElncRNAs, and DEcircRNAs. Red dots represent significantly upregulated transcripts, blue dots represent significantly downregulated transcripts, and gray dots represent transcripts without significant differential expression. Dashed lines indicate the thresholds used for differential expression analysis.

To systematically investigate the molecular mechanisms underlying *PLAG1*-mediated regulation of skeletal muscle satellite cell proliferation and differentiation, whole-transcriptome sequencing was performed on cells from the *PLAG1* knockdown group (sh*PLAG1*) and the negative control group (shNC). Principal component analysis (PCA) showed a clear separation between the sh*PLAG1* and shNC groups in principal component space, while biological replicates within each group clustered closely together (Figure 3A), indicating good reproducibility of the sequencing data. Using the predefined criteria for differential expression analysis, a total of 432 differentially expressed mRNAs (DEmRNAs; Table S2), 70 differentially expressed miRNAs (DEmiRNAs; Table S3), 163 differentially expressed lncRNAs (DElncRNAs; Table S4), and 150 differentially expressed circRNAs (DEcircRNAs; Table S5) were identified (Figure 3B). These results indicate that *PLAG1* knockdown induces extensive alterations in both coding and non-coding RNA expression profiles. Volcano plots further illustrated the overall distribution patterns of DEmRNAs (Figure 3C), DEmiRNAs (Figure 3D), DElncRNAs (Figure 3E), and DEcircRNAs (Figure 3F). Collectively, these findings suggest that *PLAG1* knockdown exerts broad effects on the transcriptomic landscape of skeletal muscle satellite cells.

### 3.4. Functional Enrichment Analysis of DEmRNAs and Validation Following PLAG1 Knockdown

**Figure 4:**
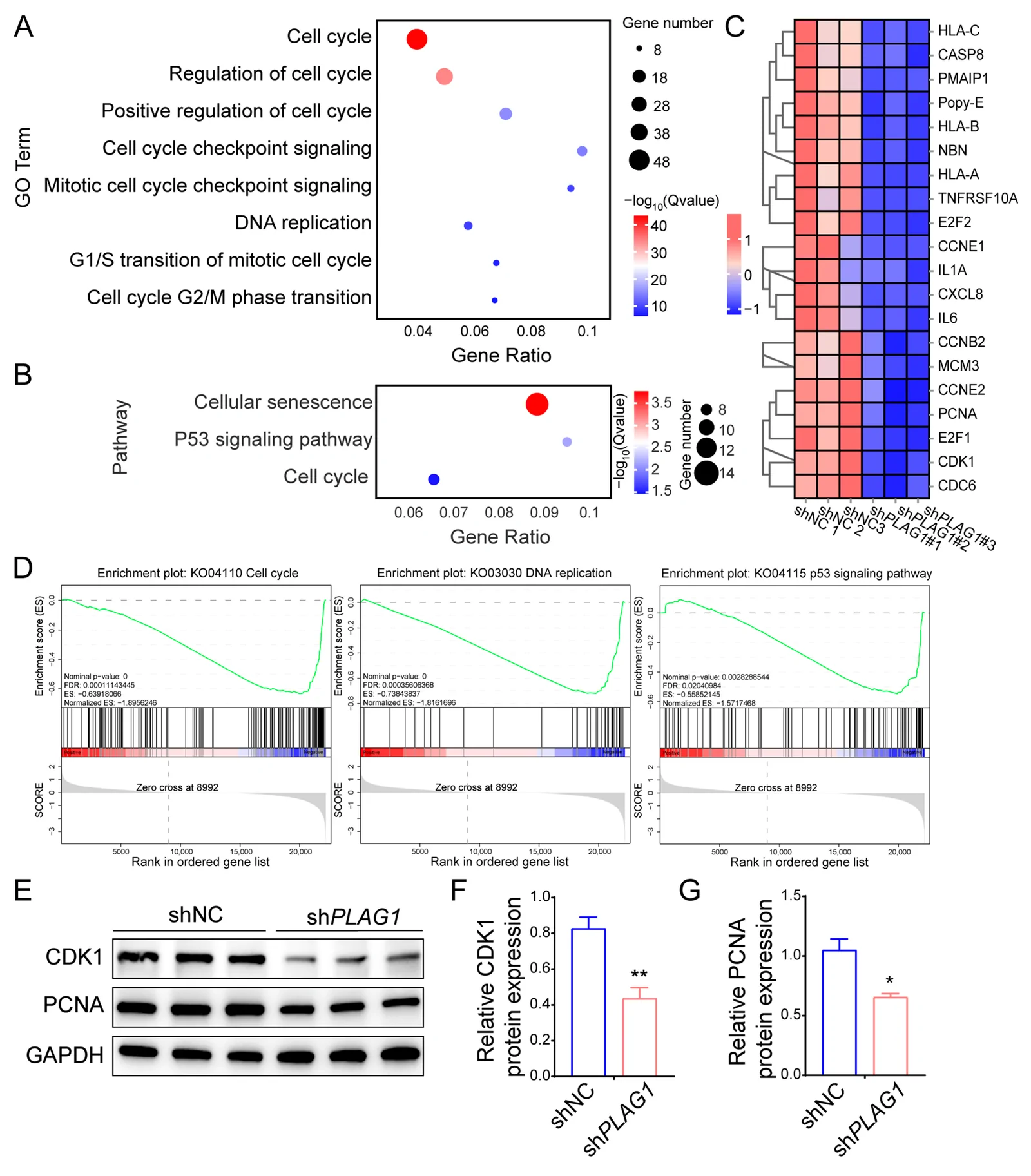
Functional enrichment analysis of DEmRNAs and validation following *PLAG1* knockdown. (**A**) GO Biological Process (GO-BP) enrichment analysis of DEmRNAs following *PLAG1* knockdown. The *x*-axis represents the Gene Ratio, bubble size indicates the number of genes enriched in each GO term, and color represents the significance level [−log10(Q-value)]. (**B**) KEGG pathway enrichment analysis of DEmRNAs following *PLAG1* knockdown. The *x*-axis represents the Gene Ratio, bubble size indicates the number of genes enriched in each pathway, and color represents the significance level [−log10(Q-value)]. (**C**) Heatmap of representative DEmRNAs enriched in the cellular senescence, p53 signaling pathway, and cell cycle. Gene expression levels were normalized using Z-scores. (**D**) Gene Set Enrichment Analysis (GSEA) of the cell cycle, DNA replication, and p53 signaling pathway gene sets. The *x*-axis represents the ranked gene list, and the *y*-axis represents the Enrichment Score (ES). The horizontal dashed line indicates ES = 0, the vertical dashed line indicates the zero-crossing point of the ranked-list metric, and the red-to-blue color bar represents the transition from positive to negative ranked-list metric values. (**E**) Western blot analysis of CDK1 and PCNA protein levels following *PLAG1* knockdown. GAPDH was used as the loading control. (**F**,**G**) Quantification of relative CDK1 (**F**) and PCNA (**G**) protein levels. Protein levels were first normalized to GAPDH and then expressed relative to the shNC control group. Data are presented as mean ± SEM. * *p* < 0.05, ** *p* < 0.01.

To systematically characterize the potential biological functions of DEmRNAs following *PLAG1* knockdown in porcine MuSCs, GO and KEGG enrichment analyses were performed. GO biological process (GO-BP) analysis revealed significant enrichment of multiple biological processes associated with cell cycle regulation (Figure 4A, Table S6). These results suggest that *PLAG1* knockdown may influence skeletal muscle satellite cell proliferation through alterations in proliferation-related regulatory networks, including cell cycle-associated pathways.

KEGG enrichment analysis further identified several significantly enriched pathways associated with cell proliferation and cell fate regulation, including the cell cycle, p53 signaling pathway, and cellular senescence (Figure 4B, Table S7). To further identify key regulatory genes, representative DEmRNAs enriched in these pathways were selected for heatmap analysis (Figure 4C). The results showed that several key cell cycle regulatory genes, including *CDK1*, *CCNB2*, *CCNE1*, *CCNE2*, *CDC6*, *PCNA*, and *MCM3*, exhibited an overall downregulated expression pattern following *PLAG1* knockdown, suggesting that *PLAG1* may regulate MuSC proliferation through cell cycle-associated regulatory networks.

In addition, Gene Set Enrichment Analysis (GSEA) demonstrated significant negative enrichment of the cell cycle, DNA replication, and p53 signaling pathway gene sets in the *PLAG1* knockdown group (Figure 4D), further indicating an overall suppression of proliferation-related biological pathways. To further validate the transcriptomic findings, the protein levels of two representative cell cycle-associated proteins, CDK1 and PCNA, were examined by Western blotting. Compared with the shNC group, *PLAG1* knockdown significantly reduced CDK1 and PCNA protein levels (Figure 4E–G), which was consistent with the transcriptomic results and further supported the involvement of proliferation-related pathways following *PLAG1* knockdown.

### 3.5. Functional Characterization of Non-Coding RNAs Following PLAG1 Knockdown

**Figure 5:**
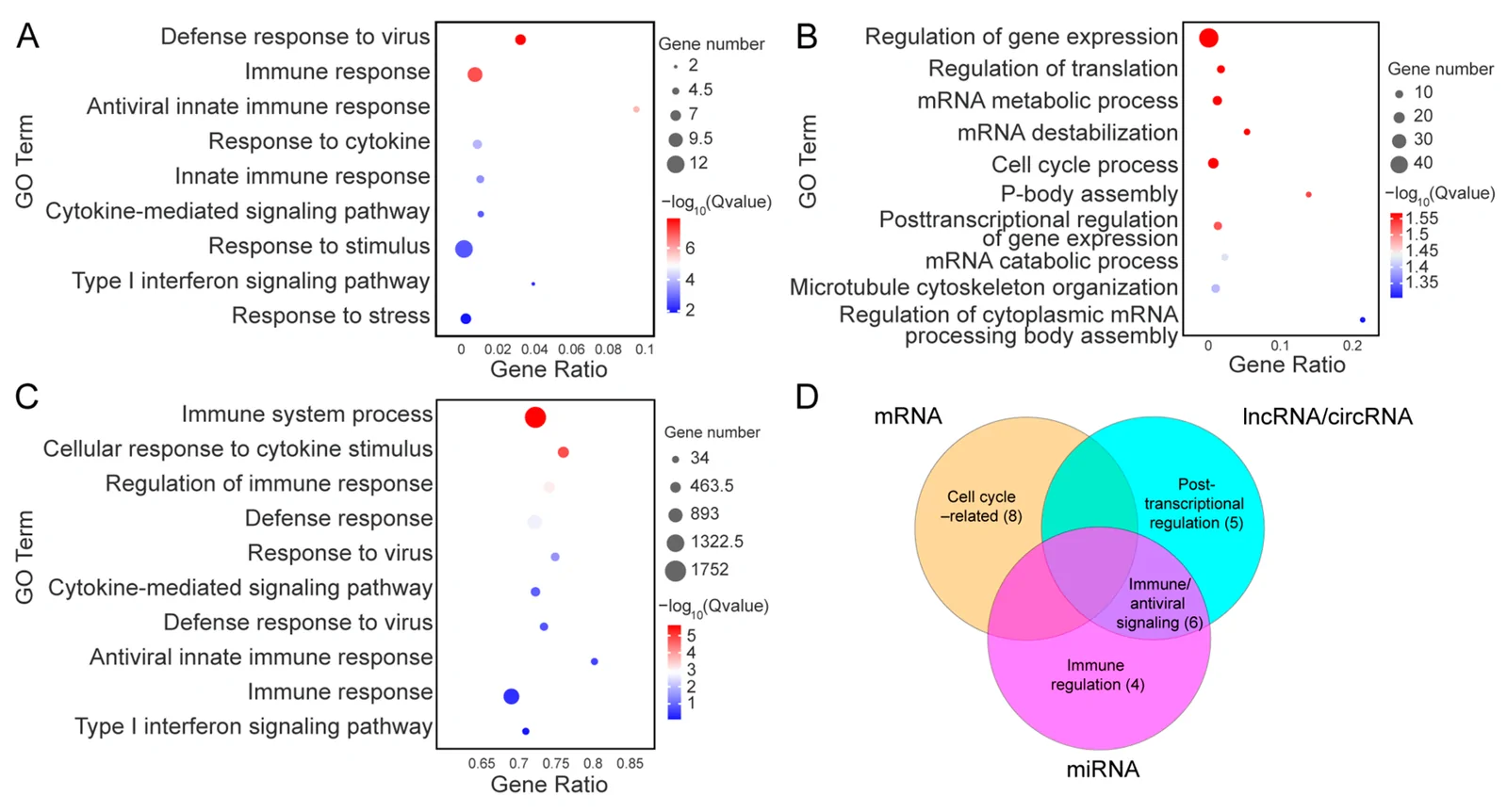
GO-BP enrichment analysis of target genes associated with differentially expressed non-coding RNAs following *PLAG1* knockdown. (**A**) GO-BP enrichment analysis of the cis target genes of DElncRNAs. The *x*-axis represents the Gene Ratio, bubble size indicates the number of target genes enriched in each GO term, and color represents the enrichment significance level [−log10(Q-value)]. (**B**) GO-BP enrichment analysis of the predicted target genes of DEcircRNAs. (**C**) GO-BP enrichment analysis of the predicted target genes of DEmiRNAs. (**D**) Venn diagram analysis of representative GO-BP terms enriched among DEmRNAs and the target genes of DElncRNAs/DEcircRNAs and DEmiRNAs. The orange, cyan, and magenta circles represent mRNAs, lncRNAs/circRNAs, and miRNAs, respectively. Numbers in parentheses indicate the number of significantly enriched GO terms within each functional category.

To further investigate the potential regulatory roles of non-coding RNAs following *PLAG1* knockdown, functional enrichment analyses were performed on the target genes of DElncRNAs, DEcircRNAs, and DEmiRNAs. GO-BP analysis of the cis target genes of DElncRNAs (Figure 5A, Table S8) revealed significant enrichment in biological processes associated with immune and stress responses. GO-BP analysis of the predicted target genes of DEcircRNAs (Figure 5B, Table S9) showed that these genes were primarily enriched in post-transcriptional regulatory processes. These findings suggest that circRNAs may contribute to the establishment of post-transcriptional regulatory networks following *PLAG1* knockdown.

Further GO-BP analysis of the predicted target genes of DEmiRNAs (Figure 5C, Table S10) showed significant enrichment in immune- and antiviral-related biological processes. In addition, several GO terms associated with cell cycle regulation and proliferation were significantly enriched, suggesting that miRNAs may participate in the regulation of skeletal muscle satellite cell proliferation.

Venn diagram analysis (Figure 5D) showed that the enriched terms of DEmRNAs were predominantly associated with cell cycle-related functions, whereas immune- and antiviral-related processes exhibited substantial overlap among the enrichment results of DElncRNA, DEcircRNA, and DEmiRNA target genes. Collectively, these findings suggest that different classes of RNAs may play distinct roles within the *PLAG1*-associated regulatory network. DEmRNAs were primarily associated with cell cycle regulation and proliferation, whereas the predicted target genes of non-coding RNAs were more closely related to immune responses, stress responses, and post-transcriptional regulatory processes.

### 3.6. ceRNA Regulatory Network Associated with PLAG1 Knockdown

To further investigate the non-coding RNA-mediated post-transcriptional regulatory relationships following *PLAG1* knockdown, ceRNA regulatory networks were constructed based on differentially expressed lncRNAs, circRNAs, miRNAs, and mRNAs, and integrated with miRNA target prediction results.

**Figure 6:**
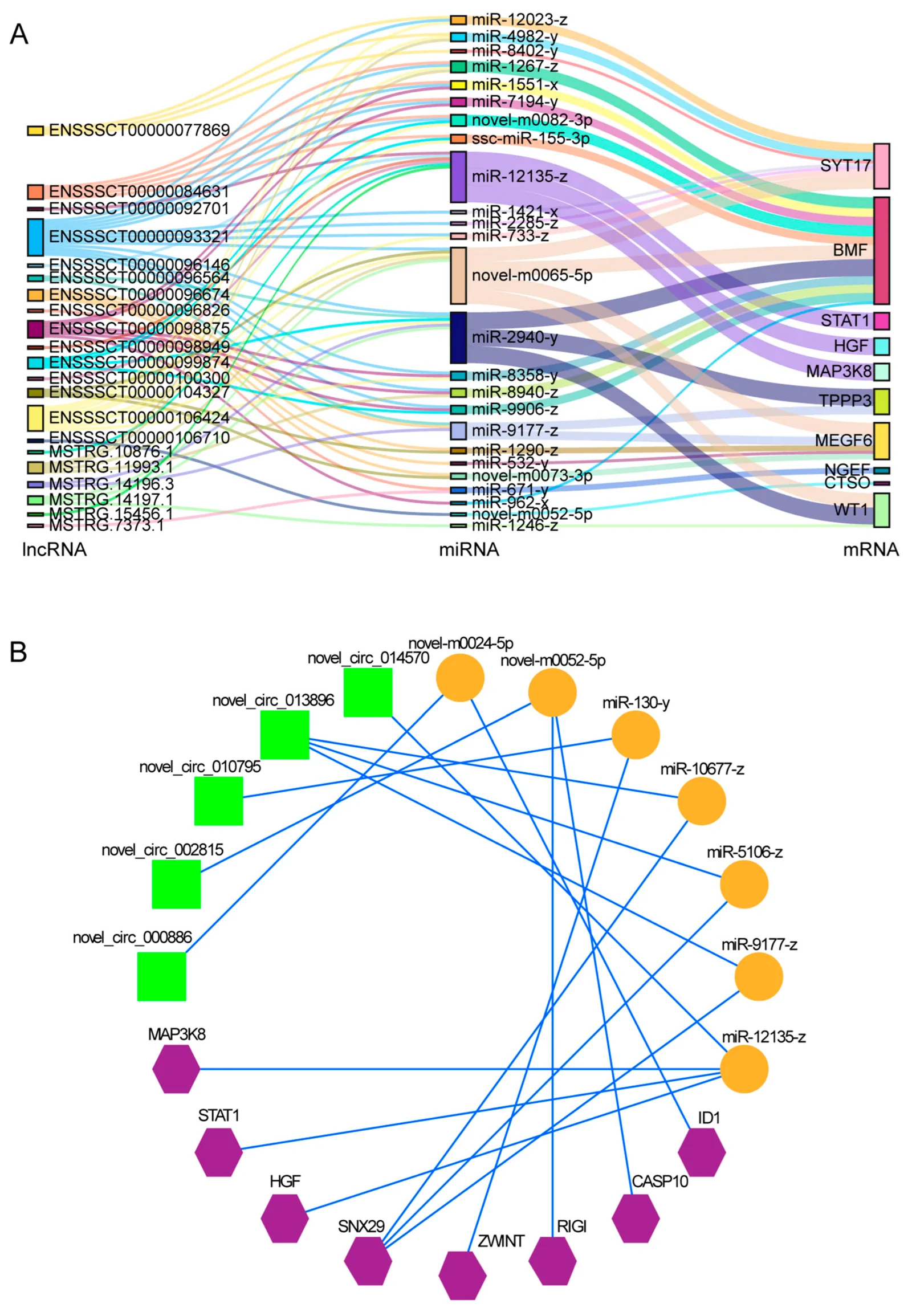
ceRNA regulatory networks based on differentially expressed non-coding RNAs following *PLAG1* knockdown. (**A**) Sankey diagram of the lncRNA–miRNA–mRNA regulatory network. DElncRNAs are shown on the left, predicted DEmiRNAs are shown in the middle, and target DEmRNAs are shown on the right. (**B**) circRNA–miRNA–mRNA regulatory network. Green squares represent DEcircRNAs, orange circles represent DEmiRNAs, and purple polygons represent target DEmRNAs.

As shown in Figure 6A, an lncRNA–miRNA–mRNA regulatory network was constructed (Table S11). Sankey diagram analysis revealed potential ceRNA regulatory relationships among multiple DElncRNAs, DEmiRNAs, and DEmRNAs. For example, miR-12135-z, miR-532-y, and miR-671-y showed extensive associations with multiple DElncRNAs and DEmRNAs and exhibited relatively high network connectivity, suggesting that they may represent potential regulatory nodes within the ceRNA network. These miRNAs were potentially associated with several candidate target genes, including *MAP3K8*, *STAT1*, *HGF*, *NGEF*, and *MEGF6*.

The circRNA–miRNA–mRNA regulatory network is shown in Figure 6B (Table S12). The results revealed potential regulatory associations among multiple circRNAs, miRNAs, and their candidate target genes. In particular, circRNAs such as novel_circ_014570, novel_circ_013896, and novel_circ_010795 were predicted to interact with miR-12135-z, miR-9177-z, miR-5106-z, and miR-130-y and were linked to candidate target genes including *MAP3K8*, *STAT1*, *HGF*, *SNX29*, *CASP10*, and *ID1*, thereby forming multiple potential circRNA–miRNA–mRNA regulatory relationships.

### 3.7. qRT-PCR Validation of Representative RNAs Following PLAG1 Knockdown

**Figure 7:**
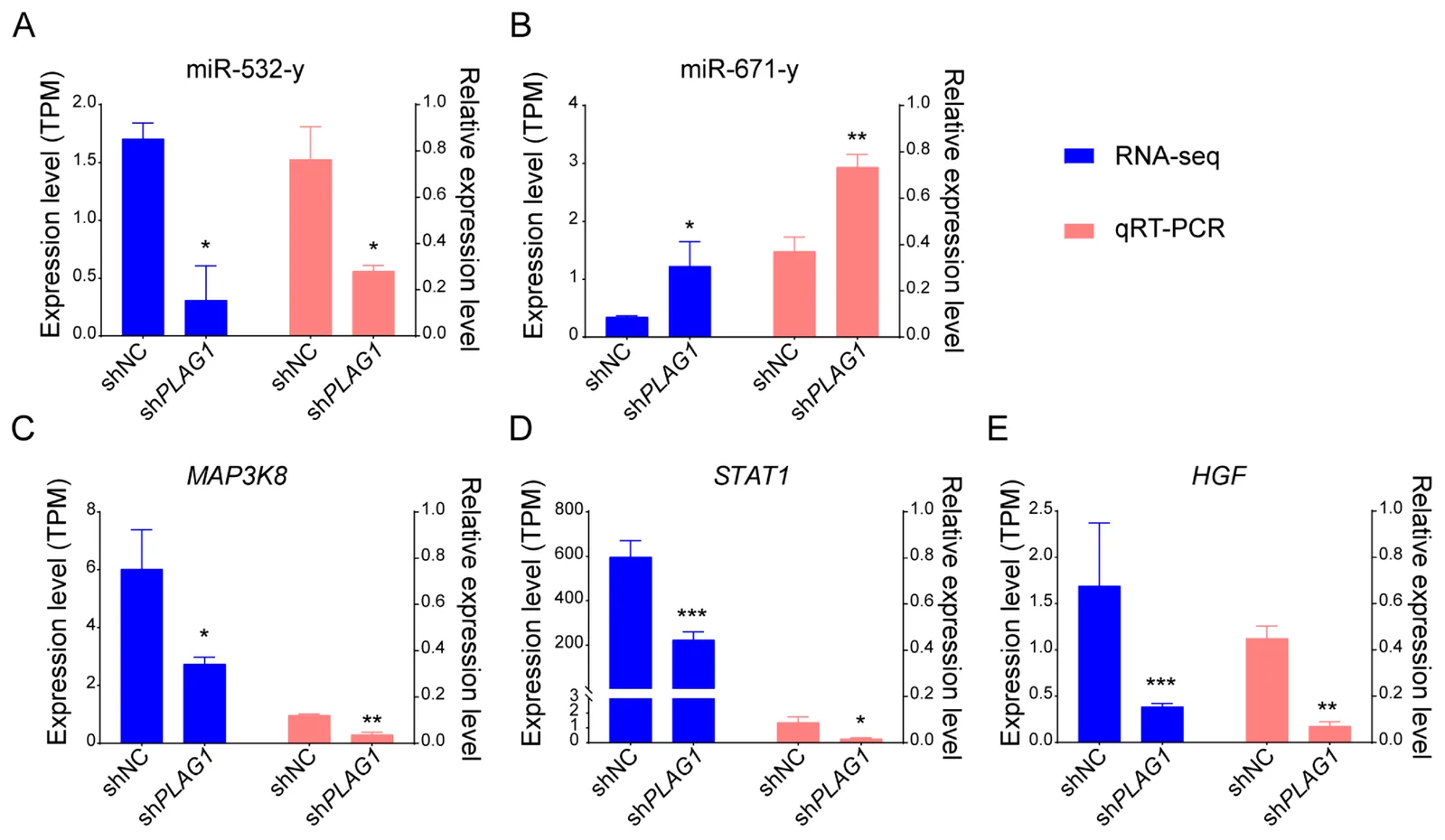
qRT-PCR validation of representative RNAs following *PLAG1* knockdown. (**A**,**B**) Validation of representative differentially expressed miRNAs, including miR-532-y (**A**) and miR-671-y (**B**). (**C**–**E**) Validation of representative differentially expressed mRNAs, including *MAP3K8* (**C**), *STAT1* (**D**), and *HGF* (**E**). Blue bars represent transcriptome sequencing data, and pink bars represent qRT-PCR results. Expression levels from sequencing data are presented as TPM values. Data are presented as mean ± SEM (*n* = 3). * *p* < 0.05, ** *p* < 0.01, *** *p* < 0.001.

To further validate the reliability of transcriptomic analysis, representative differentially expressed RNAs were selected for qRT-PCR analysis. The results showed that the expression patterns of the selected RNAs were consistent with the transcriptomic analysis results, supporting the reliability of the transcriptomic sequencing data. Specifically, miR-532-y was downregulated, whereas miR-671-y was upregulated following *PLAG1* knockdown. In addition, *MAP3K8*, *STAT1*, and *HGF* exhibited significantly decreased expression levels in the *PLAG1* knockdown group compared with the shNC control group (Figure 7).

## 4. Discussion

This study systematically investigated the expression pattern, regulatory role, and potential molecular regulatory mechanisms of the transcription factor *PLAG1* in Sujiang pig MuSCs. The results demonstrated that *PLAG1* expression was continuously upregulated during satellite cell differentiation and that *PLAG1* promoted both satellite cell proliferation and differentiation, supporting the role of *PLAG1* in regulating MuSC function. Furthermore, integrated whole-transcriptome analysis revealed that *PLAG1* knockdown induced significant alterations in the expression profiles of cell cycle-related genes and multiple non-coding RNAs, suggesting that *PLAG1* may participate in MuSC regulation through cell cycle-related pathways and potentially associated non-coding RNA regulatory networks. These findings provide further insights into the role of *PLAG1* in porcine MuSC regulation.

*PLAG1* contains a typical C2H2 zinc-finger DNA-binding domain and a transcriptional activation domain. Phylogenetic analysis demonstrated that *PLAG1* is highly conserved among mammals. Previous studies have demonstrated that *PLAG1* plays important roles in growth regulation and is associated with economically important traits in livestock. For example, *PLAG1* has been identified as a candidate gene affecting body size and growth-related traits in cattle and pigs [20,25,26]. At the molecular level, *PLAG1* can regulate growth factor expression by activating IGF2 transcription through promoter binding [27]. In skeletal muscle-related studies, *PLAG1* has been reported to promote the proliferation of bovine primary myoblasts through the PI3K–Akt signaling pathway and is subject to regulation by miR-1 [28]. However, the regulatory role and molecular mechanisms of *PLAG1* in porcine MuSCs remain unclear. In the present study, functional assays demonstrated that *PLAG1* positively regulated porcine MuSC proliferation and differentiation. In addition, whole-transcriptome analysis revealed that *PLAG1* knockdown affected the expression of genes involved in cell cycle regulation and multiple non-coding RNAs, suggesting that *PLAG1* may influence MuSC function through transcriptional regulation and potentially associated non-coding RNA-mediated regulatory networks.

Transcriptomic GO enrichment analysis revealed that multiple biological processes associated with cell cycle regulation were significantly enriched following *PLAG1* knockdown, suggesting a potential role of *PLAG1* in the regulation of cell cycle progression. The proliferation of skeletal muscle satellite cells depends on precise control of the cell cycle, in which the G1/S and G2/M transitions represent critical checkpoints that determine whether cells enter DNA replication and mitosis. Further analysis showed that several key cell cycle regulatory genes, including *CDK1*, *CCNB2*, *CCNE1*, *CCNE2*, *CDC6*, *PCNA*, and *MCM3*, exhibited an overall downregulated expression pattern following *PLAG1* knockdown. These genes are involved in DNA replication initiation, cell cycle progression, and mitotic regulation. Therefore, the decreased expression of these genes may impair the proliferative capacity of MuSCs by limiting cell cycle progression and DNA replication. Consistent with these transcriptomic findings, functional assays demonstrated that *PLAG1* overexpression significantly promoted MuSC proliferation, whereas *PLAG1* knockdown exerted the opposite effect. Furthermore, the reduced protein levels of CDK1 and PCNA following *PLAG1* knockdown provide additional evidence supporting the involvement of cell cycle-associated regulation in *PLAG1*-mediated control of MuSC proliferation. KEGG enrichment analysis and GSEA both identified significant alterations in the p53 signaling pathway following *PLAG1* knockdown. As an important regulator of cell cycle checkpoints and cellular stress responses [29,30], p53 signaling may contribute to the cellular changes induced by *PLAG1* deficiency. However, whether *PLAG1* directly regulates p53 signaling or affects this pathway through downstream regulatory networks requires further investigation.

Notably, although functional experiments demonstrated that *PLAG1* influences satellite cell differentiation, transcriptomic enrichment analyses did not identify significantly enriched myogenesis-related pathways. This observation suggests that the effect of *PLAG1* on differentiation may not be primarily mediated through direct regulation of the myogenic transcriptional network but rather through modulation of cell cycle status, thereby influencing the transition of satellite cells from proliferation to differentiation. Cell cycle exit is an essential prerequisite for myogenic differentiation, indicating that *PLAG1*-mediated regulation of cell cycle progression may influence the transition of satellite cells toward differentiation. In addition, by promoting satellite cell proliferation, *PLAG1* may increase the number of myogenic cells available for fusion during differentiation, thereby facilitating myotube formation and maturation. Consistent with this hypothesis, *PLAG1* overexpression enhanced cell proliferation and increased both the differentiation index and the number of nuclei per myotube in the present study. Therefore, the promotive effect of *PLAG1* on skeletal muscle satellite cell differentiation may, at least in part, depend on its regulatory role in cell proliferation. However, the specific molecular mechanisms through which *PLAG1* regulates myogenic differentiation require further investigation.

Previous studies have demonstrated that miRNAs play important roles in skeletal muscle development by regulating target gene expression at the post-transcriptional level [31,32]. Several muscle-related miRNAs, including miR-1, miR-206, and miR-181a, have been shown to regulate satellite cell proliferation and differentiation through modulation of myogenic genes [33,34]. In the present study, whole-transcriptome analysis revealed significant alterations in the expression of multiple miRNAs following *PLAG1* knockdown, including miR-532-y and miR-671-y, which were further validated by qRT-PCR. These findings suggest that these miRNAs may represent potential regulatory factors associated with *PLAG1*-mediated changes in MuSCs. However, their specific biological functions and underlying molecular mechanisms require further investigation and experimental validation.

According to the ceRNA hypothesis, lncRNAs and circRNAs can competitively bind miRNAs, thereby alleviating their inhibitory effects on target mRNAs and participating in complex post-transcriptional regulatory networks involved in various biological processes, including cell proliferation and differentiation [35]. In this study, a ceRNA network was constructed by integrating differentially expressed lncRNAs, circRNAs, miRNAs, and mRNAs following *PLAG1* knockdown. Network analysis identified miR-12135-z as a candidate regulatory miRNA and predicted potential regulatory associations with several candidate target genes, including *MAP3K8*, *STAT1*, and *HGF*. The expression changes in these candidate genes were further validated by qRT-PCR, supporting their association with molecular alterations following *PLAG1* knockdown. However, the direct regulatory relationships between miR-12135-z and these candidate target genes require further experimental validation. Therefore, the ceRNA network constructed in this study should be considered a potential regulatory framework that provides candidate molecular clues for further investigation of *PLAG1*-associated non-coding RNA regulation.

### Limitations

This study has several limitations. First, all functional experiments were performed using cultured porcine skeletal muscle satellite cells. Therefore, the present findings support a role for *PLAG1* in regulating MuSC proliferation and differentiation in vitro but do not directly demonstrate its effects on muscle growth, carcass traits, or other economically important traits at the animal level. Further in vivo functional studies and large-scale population analyses are required before the potential application of *PLAG1* in pig breeding can be evaluated. Second, the non-coding RNA regulatory relationships identified within the ceRNA network were mainly predicted through bioinformatic analyses, and the proposed molecular interactions require further validation using approaches such as dual-luciferase reporter assays and RNA immunoprecipitation. In addition, the downstream signaling pathways and molecular mechanisms mediated by *PLAG1* remain to be further elucidated. Third, only female piglets were included in this study. Therefore, whether the present findings are equally applicable to male pigs remains to be determined, and future studies involving both sexes are required. Although these limitations may restrict the broader interpretation and generalizability of the findings, they are unlikely to affect the main conclusions of this study regarding the regulatory role of *PLAG1* in porcine MuSC proliferation and differentiation.

## 5. Conclusions

In conclusion, this study provides new insights into the regulatory role of *PLAG1* in porcine MuSCs by integrating functional assays and transcriptomic analyses. Functional experiments demonstrated that *PLAG1* promoted MuSC proliferation and differentiation. Whole-transcriptome analysis further revealed that *PLAG1* knockdown altered the expression patterns of genes involved in cell cycle regulation and the p53 signaling pathway, as well as multiple non-coding RNAs. Moreover, ceRNA network analysis identified potential regulatory relationships among lncRNAs, circRNAs, miRNAs, and mRNAs associated with *PLAG1* function. Collectively, these findings contribute to a better understanding of the molecular regulation of porcine MuSCs and provide further insights into the role of *PLAG1* in satellite cell regulation. Future studies should further validate the predicted ceRNA regulatory relationships, elucidate the downstream molecular mechanisms of *PLAG1*, and evaluate its regulatory role using in vivo models.

## Data Availability

The data presented in this study are openly available in [NCBI] [https://www.ncbi.nlm.nih.gov/bioproject/PRJNA1477753/; accessed on 12 June 2026] [PRJNA1477753].

## Supplementary Materials

The following supporting information can be downloaded at https://www.mdpi.com/article/10.3390/vetsci13080734/s1. Table S1: Species and their NCBI RefSeq accession numbers for *PLAG1* mRNA used in this study. Figure S1: Original Western blot images corresponding to Figure 4E. Table S2: Group_shNC-vs-shPLAG1_DEmRNA_significant_anno. Table S3: Group_shNC-vs-shPLAG1_DEmiRNA_significant_anno. Table S4: Group_shNC-vs-shPLAG1_DElncRNA_significant_anno. Table S5: Group_shNC-vs-shPLAG1_DEcircRNA_significant_anno. Table S6: DEmRNA_GO_BP_Group_shNC-vs-shPLAG1. Table S7: DEmRNA_KEGG_Group_shNC-vs-shPLAG1. Table S8: DElncRNA_cis_GO_BP_Group_shNC-vs-shPLAG1. Table S9: DEcircRNA_GO_BP_Group_shNC-vs-shPLAG1. Table S10: DEmiRNA_GO_BP_Group_shNC-vs-shPLAG1. Table S11: lncRNA-mRNA.ceRNA. Table S12: circRNA-mRNA.ceRNA.

## Abbreviations

The following abbreviations are used in this manuscript:

MuSCs	Skeletal muscle satellite cells
PLAG1	Pleomorphic adenoma gene 1
ORF	Open reading frame
ceRNA	Competing endogenous RNA
GO	Gene Ontology
KEGG	Kyoto Encyclopedia of Genes and Genomes
MOI	Multiplicity of infection
PCA	Principal component analysis
DE	Differentially expressed
