# Supplementary material for: PLAG1 Promotes Proliferation and Differentiation of Sujiang Pig MuSCs: Insights from Whole-Transcriptome and ceRNA Network Analyses
Source: Vet Sci. 2026 Jul 24;13(8):734. doi: 10.3390/vetsci13080734 (PMC13517130; doi:10.3390/vetsci13080734)
Supplement: Supplementary file 1 [file vetsci-13-00734-s001.zip › Figure S1.pdf]

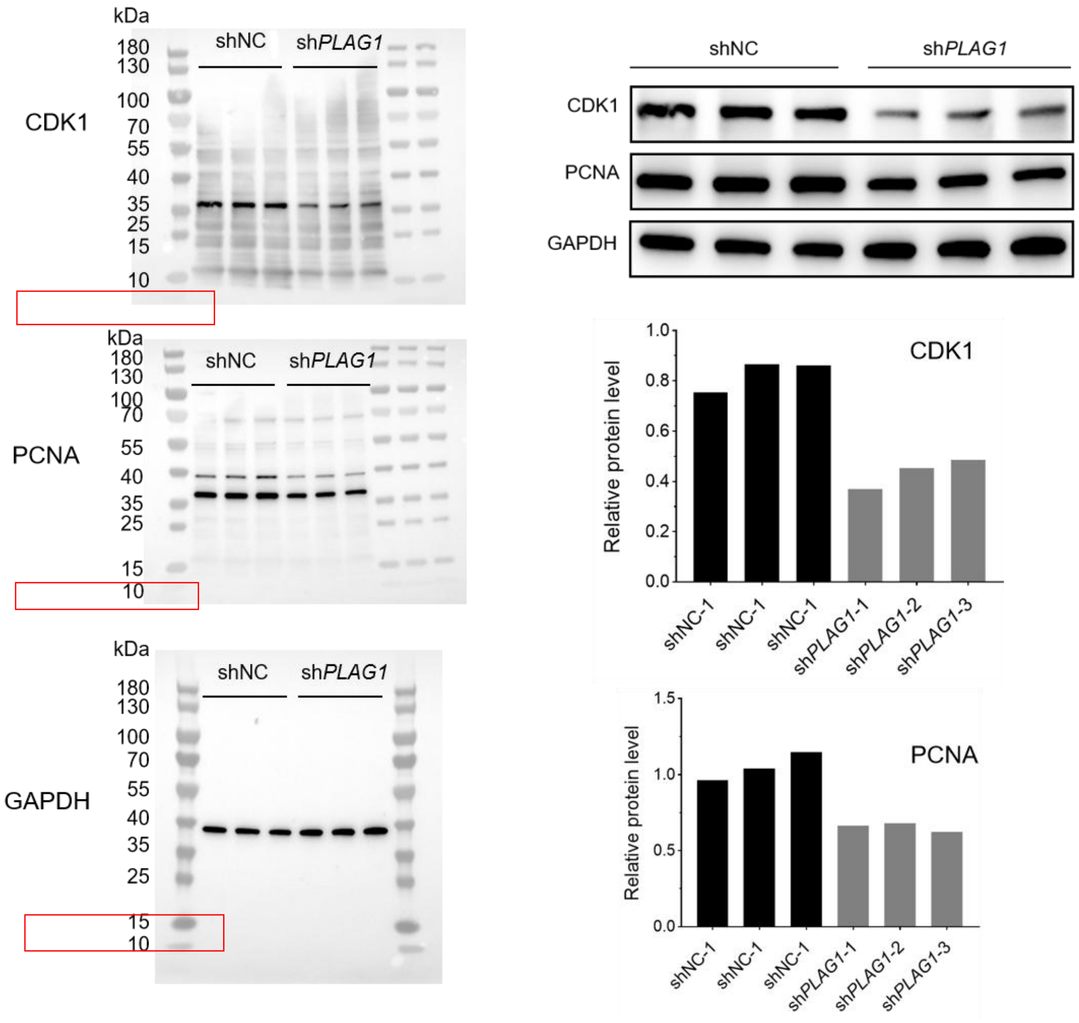

Figure S1: Original Western blot images corresponding to Figure 4E.
