# Supplementary material for: PLAG1 Promotes Proliferation and Differentiation of Sujiang Pig MuSCs: Insights from Whole-Transcriptome and ceRNA Network Analyses
Source: Vet Sci. 2026 Jul 24;13(8):734. doi: 10.3390/vetsci13080734 (PMC13517130; doi:10.3390/vetsci13080734)
Supplement: Supplementary file 1 [file vetsci-13-00734-s001.zip › Table S1.pdf]

Table S1. Species and their NCBI RefSeq accession numbers for *PLAG1* mRNA used in this study.

Species	NCBI RefSeq accession number
Homo sapiens (human)	NM_002655.3
Sus scrofa (pig)	XM_013996756.2
Mus musculus (house mouse)	NM_019969.3
Bos taurus (domestic cattle)	XM_005215432.4
Ovis aries (sheep)	XM_012183720.3
Capra hircus (goat)	XM_013969222.2
Gallus gallus (chicken)	NM_001031577.2
Rattus norvegicus (Norway rat)	NM_001008316.3
Macaca mulatta (rhesus monkey)	XM_001085496.5
Anas platyrhynchos (mallard)	XM_038175435.2
Anser cygnoides (swan goose)	XM_066993006.1
Xenopus tropicalis (tropical clawed frog)	XM_004915191.4
Danio rerio (zebrafish)	NM_001039814.1
